# The Impact of Trust in Science on COVID-19 Vaccine Attitudes: Parallel Mediation Through Conspiracy Beliefs and General Vaccine Hesitancy

**DOI:** 10.5152/eurasianjmed.2025.251024

**Published:** 2025-11-25

**Authors:** Sümeyye Kara, Serra Sevde Hatipoğlu, Nesibe Zeynep Arslanoğlu, Zahide Erdoğan

**Affiliations:** 1 Department of Sociology and Social Policy, University of Leeds School of Social Science, Leeds, UK; 2 Department of Sociolgy, Ankara Hacı Bayram Veli University Faculty of Letters, Ankara, Türkiye

**Keywords:** Conspiracy beliefs, COVID-19 vaccine attitudes, process model 4, trust in science, vaccine hesitancy

## Abstract

**Background:**

This study examines the impact of trust in science on individuals’ attitudes toward the COVID-19 vaccine, with an application to the parallel mediating roles of belief in conspiracy theories and general vaccine hesitancy.

**Methods:**

A survey of 469 adults in Türkiye was conducted online and paper-based. Direct and indirect effects (IEs) were estimated by parallel mediation analysis using PROCESS Macro Model 4 with 5000 bootstrap resamples.

**Results:**

Trust in science was found to influence COVID-19 vaccine attitudes indirectly through 2 distinct psychological mechanisms: reduced belief in conspiracy theories and more positive general vaccine attitudes. Both indirect pathways were statistically significant, confirming their mediating roles. Although the mediation effect through general vaccine attitudes was larger in magnitude, the difference between the 2 IEs was not statistically significant.

**Conclusion:**

These results point out 2 separate psychological routes connecting scientific confidence to vaccination acceptance. General vaccine attitudes could be more profound and lasting than crisis-specific ones. This paper provides theoretical and practical insights for creating long-term public health strategies that foster trust and combat both misinformation and deep-seated vaccine skepticism by using a parallel mediation approach in the sociocultural setting of Türkiye.

**Main Points**

- As demonstrated by parallel mediation analysis, trust in science significantly predicted attitudes toward the COVID-19 vaccine indirectly through conspiracy beliefs and general vaccine hesitancy.
- General vaccine hesitancy exhibited a more pronounced indirect effect than conspiracy beliefs, indicating a stronger mediating role in shaping vaccine acceptance.
- The difference between the 2 mediators was not statistically significant, suggesting both belief systems exert comparable influence.
- Belief in conspiracy theories correlated negatively with trust in science and positively with vaccine hesitancy, highlighting its role as a psychological barrier.
- These findings emphasize the need to target general vaccine attitudes in long-term public health strategies.

## Introduction

Vaccine hesitancy has become the main concern in global public health, especially during the COVID-19 pandemic. Even though COVID-19 is no longer considered a global health emergency by the World Health Organization (WHO), its impact on vaccine acceptance persists. A growing body of literature documents widespread hesitancy across diverse populations.^1^^-^^5^ Previous research has reported high levels of vaccine hesitancy from different countries. Particularly, cross-cultural research shows that Turkiye reported the highest level of vaccine hesitancy during the COVID-19 pandemic.^1^^,^^6^ Therefore, it is important to focus on Türkiye and examine the factors influencing COVID-19 vaccine hesitancy.

Based on literature, distrust of science and scientists and belief in conspiracy theories have been reported to be highly correlated with COVID-19 vaccine hesitancy and general vaccine hesitancy. While the pandemic elevated the visibility of science, it also amplified misinformation, conspiracy theories, and distrust in scientific institutions in the post-truth era. Trust in science has been consistently linked to adherence to public health measures and vaccine uptake.^4^^,^^6^^,^^7^ Previous research has found that post-truth discourse undermines the authority of the scientific community in shaping knowledge and negatively impacts individuals’ trust in science. This leads to the development of negative emotions toward vaccines among individuals, with these emotions playing a more decisive role in vaccine acceptance than statistical evidence. Anti-vaccine arguments strengthen resistance to scientific knowledge and its products like vaccines, aligning with the core values of individuals’ mindsets.^8^ Fear disconnected from reality becomes widespread and more contagious in situations of uncertainty, such as pandemics. Since these beliefs contain ideas that contradict contemporary scientific theories, data on the safety and efficacy of COVID-19 vaccination contributes to fueled vaccine hesitancy.^7^

Yet, this trust has weakened for many individuals, coinciding with increased belief in conspiracy theories,^9^ which in turn can abrade public confidence and contribute to vaccine resistance.^10^ Studies have demonstrated that various conspiracy theories have been developed regarding the COVID-19 vaccine, and a tendency to believe in these theories has been observed even among vaccinated individuals.^11^ An examination of the intensity of endorsement of conspiracy theories related to the coronavirus vaccine revealed that a significant majority of participants believe the virus was manufactured in a laboratory.^12^ Among the conspiracy theories in the context of the COVID-19 vaccine, the claim that vaccinating children is harmful and that this information is being concealed represents the least widely accepted theory, whereas the notion that vaccine companies are hiding the vaccine’s harms constitutes the most widely accepted conspiracy theory.^10^ Furthermore, an increase in belief in conspiracy theories has been identified to strongly correlate with a reduction in vaccination rates.^13^

Türkiye reflects similar dynamics. Even before the pandemic, childhood vaccination rates declined from 80% to 74%,^14^ and post-pandemic trends indicate persistent hesitancy.^8^ Qin et al^15^conducted a cross-sectional survey to evaluate pandemic fatigue and vaccine hesitancy in China in the post-pandemic era. The findings emphasize that people with high pandemic fatigue might be prone to hesitate to receive the next dose of the vaccine. This decline in vaccination uptake is often attributed to the rise of conspiracy beliefs and a decreasing trust in scientific authority.^16^ It is also noteworthy that a considerable minority endorses a conspiratorial mindset related to general vaccines, while an even larger proportion remains ambivalent regarding the veracity of such beliefs.^10^

As of current global data, 70.5% of the world population has received at least 1 dose of a COVID-19 vaccine. Data from the Turkish Ministry of Health indicates that 93% of the population in Türkiye has received at least 1 vaccine dose, while the rate for those who have received 2 doses drops to 85%. In certain provinces located in the eastern and southeastern regions of the country, this rate declines further, reaching as low as 63%.^17^ The decline in vaccination rates is noteworthy despite the high transmissibility and mortality rates of the virus.

Based on the literature, this study argues that the relationship between COVID-19 vaccine hesitancy and trust in science and scientists is mediated by general vaccine attitudes and belief in conspiracy theories. This study examines, with regard to the post-COVID-19 era, whether trust in science and scientists influences attitudes toward the COVID-19 vaccine and whether this relationship is mediated by conspiracy beliefs and general vaccine hesitancy. It also evaluates the relative strength of these indirect effects (IEs).

### Literature Review and Conceptual Framework

Vaccine hesitancy, defined as a “delay in acceptance or refusal of vaccination despite availability,” is a complex, context-dependent phenomenon influenced by individual, cultural, and political factors.^18^ In the literature, while demographic variables like age, gender, education, and income are frequently associated with hesitancy, emerging research emphasizes the central role of trust in science, belief in conspiracy theories, and general vaccine hesitancy.

In this study, the trust in science and scientist variables have been proposed to explain COVID-19 vaccine hesitancy as an independent variable; belief in conspiracy theories and general vaccine hesitancy are considered to mediate the relationship between these 2 variables.

#### Trust in Science and Scientists

Vaccine hesitancy has become increasingly widespread, driven by distrust in science, the pursuit of alternative knowledge, and post-truth discourses. The strong relationship between these factors suggests that vaccine hesitancy is closely associated with distrust in science and commonly accepted scientific knowledge. As vaccine hesitancy grows among individuals, an increase in the prevalence of those who embrace alternative beliefs and conspiracy theories is anticipated.^4^^,^^8^ Therefore, trust in science and scientists emerges as one of key factors influencing vaccine acceptance, particularly during periods of uncertainty such as pandemics. Eslen-Ziya and Pehlivanli (2022) argue that in the post-truth era, the rise of alternative knowledge stems from a crisis of truth and scientific authority. Drawing on the emotional-eco-chamber theory, they emphasize that the rapid circulation of emotion accelerates the uptake of alternative knowledge, often at the expense of scientific evidence. This emotional mediation weakens scientific authority, as personal justifications increasingly shape public opinion.^8^ Individuals with lower trust in science and scientists exhibit hesitancy in accepting scientific knowledge and products such as vaccines.^3^^,^^7^ For instance, despite the absence of scientific evidence, a high proportion of people believe that vaccines are linked to autism. Studies indicate that individuals who have greater trust in science and scientists are more prone to accept vaccines and adhere to virus prevention measures.^4^^,^^6^^,^^7^^,^^19^ In this context, trust in science and scientists has a significant role in explaining the high levels of hesitancy toward the COVID-19 vaccine.

However, confidence in science and vaccine hesitancy do not always demonstrate a linear relationship. Cross-cultural findings indicate that context matters. For example, Turkish respondents have greater trust in science than Americans, yet show lower vaccine acceptance.^1^^,^^6^ While a strong negative relationship between trust in science and conspiracy beliefs was observed in Ukraine and Germany, this pattern was not found in Türkiye.

These inconsistencies highlight the importance of examining mediating mechanisms. This study proposes that both conspiracy beliefs and general vaccine hesitancy mediate the effect of trust in science on COVID-19 vaccine attitudes. It also investigates which of these mediators exerts a stronger IE. By focusing on the interrelations among these variables, rather than treating them in isolation, this research offers a more nuanced insight into vaccine decision-making processes.

H1: Trust in science and scientists positively affect individuals’ attitudes toward the COVID-19 vaccine through belief in conspiracy theories.

#### General Vaccine Hesitancy

Throughout the global health crisis of COVID-19, the anti-vaccine movement has become increasingly prominent, a concern that was already recognized by WHO in 2019, which listed it among the 10 global health threats (WHO, 2019). A systematic review by Dubé indicates that decision-making regarding vaccination is a complex process influenced by emotional, cultural, social, and regional factors.^20^ Notably, trust in health professionals and institutions, and governments, has been found to be closely linked to vaccination behavior.

Prior vaccination behavior serves as a dependable indicator of COVID-19 vaccine acceptance. Individuals who have received the influenza vaccine or vaccinated their offspring are typically more inclined to accept COVID-19 vaccination.^3^ General distrust in vaccines, previous negative vaccination experiences, or unverified misconceptions about vaccines have been found to increase COVID-19 vaccine hesitancy.^3^^,^^21^

H2: Trust in science and scientists positively affect individuals’ attitudes toward the COVID-19 vaccine through general anti-vaccine attitudes.

#### Conspiracy Theories

“‘Conspiracy theories are attempts to explain the ultimate causes of significant social and political events and circumstances with claims of secret plots by 2 or more powerful actors.”^22^ As the coronavirus pandemic began to spread rapidly, misinformation and conspiracy theories about the virus also proliferated as a viral phenomenon. This ambiguous period facilitated individuals’ belief in conspiracy theories and increased the likelihood of adopting vaccine-hesitant or vaccine-refusal attitudes.

Empirical studies conducted in Türkiye, both prior to and during the pandemic, confirm a negative association between conspiracy beliefs and vaccine acceptance.^13^^,^^16^^,^^23^ Findings indicate that approximately 48% of individuals in Türkiye endorse at least 1 conspiracy theory related to the virus.^24^^,^^25^ Also, a systematic review covering 39 studies supports this conclusion.^2^

In the United States, being young^26^ and in Türkiye, being over the age of 60^24^ are associated with higher belief in conspiracy theories. Additionally, low levels of education and income,^23^^,^^26^^,^^27^ being female,^25^ and attributing high importance to religion^27^ have also been linked to increased susceptibility to conspiratorial thinking. A conspiratorial mindset is recognized as a significant determinant of vaccine hesitancy.^9^ Furthermore, certain personality traits such as social dominance orientation and authoritarianism, as well as high spirituality, elevated narcissism, and low analytical thinking, have been shown to predict belief in conspiracy theories.^27^^-^^29^

Beliefs suggesting that COVID-19 was engineered for control or disseminated through technology diminish trust in public health institutions and scientific authority.^9^^,^^10^ Among various conspiracy theories, Turkish participants predominantly are convinced that the coronavirus was man-made in a laboratory;^12^ 29% of Americans similarly believe the virus was either deliberately or accidentally created in a laboratory setting,^26^ while British participants are most likely to believe that vaccine companies conceal the harmful effects of vaccines.^10^ In the UK, the primary reason for vaccine hesitancy is belief in both general and vaccine-specific conspiracy theories.^10^

According to a comparative study involving participants from the UK, US, and Türkiye, belief in conspiracy theories is most prevalent among Turkish respondents, followed by Americans. A one-point increase in belief in COVID-19 conspiracy theories is associated with a reduction in vaccine acceptance by 89% in the UK, 127% in the US, and 32% in Türkiye. Similarly, a one-point increase on the Conspiracy Mentality Scale corresponds to a decrease in vaccine acceptance by 82%, 25%, and 72%, respectively.^6^

However, this association is not deterministic. Some vaccine-hesitant individuals hold conspiracy beliefs similar to those of vaccinated individuals, or they may simply lack the motivation to seek reliable information.^4^^,^^30^ Another study suggests that decreasing willingness to be vaccinated can also lead to increased endorsement of conspiracy theories.^30^

H3: Trust in science and scientists affects attitudes toward the COVID-19 vaccine through belief in conspiracy theories.

This study contributes to the literature by exploring not only the effects of these variables but also their interrelationship. Specifically, it proposes that the extent to which trust in science reduces vaccine hesitancy depends on individuals’ susceptibility to conspiratorial thinking and their broader attitudes toward vaccines.

H4: There is a statistically significant difference between the IE mediated by conspiracy beliefs and the IE mediated by general vaccine hesitancy.

**Figure 1.**
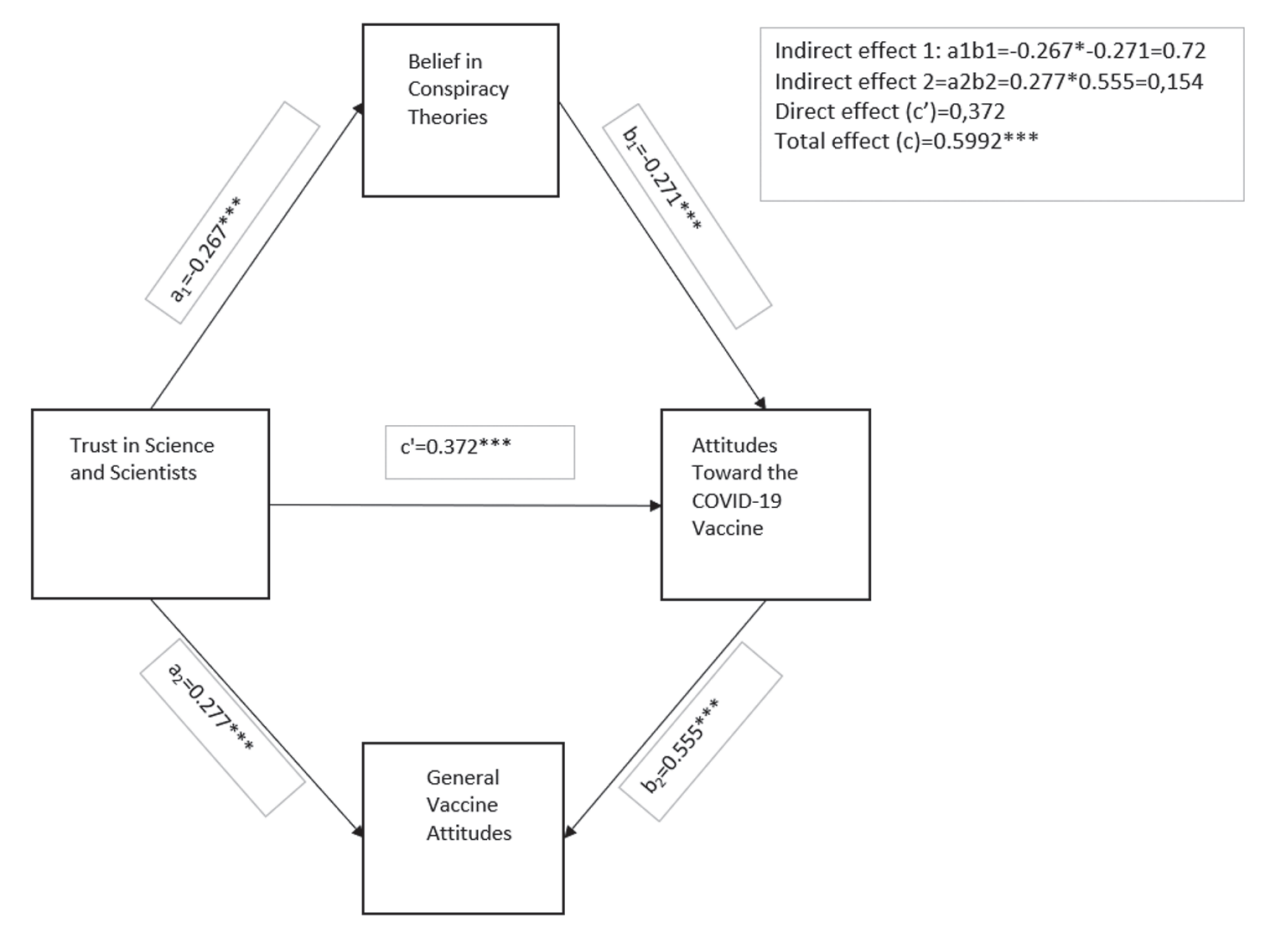
Parallel mediation model predicting attitudes toward the COVID-19 vaccine. *P < .05, **P < .01, ***P < .001.

The proposed model is depicted in Figure 1.

## Materials and Methods

### Type of the Study

This study employs a quantitative survey design to examine the mediating roles of conspiracy beliefs and general vaccine attitudes in the relationship between trust in science and COVID-19 vaccine acceptance. The research focuses on the Turkish population and uses mediation analysis to evaluate the hypothesized IEs.

### Place and Time of the Study

The study was conducted in Türkiye between February and April 2024.

### Participants

The survey was conducted among adults aged 18 and above residing in Türkiye, utilizing a snowball sampling strategy through the researchers’ professional and social networks. Of the 469 individuals who participated, 356 completed the questionnaire online, while 113 responded via paper-based forms. The sample was predominantly female (63.3%), with the largest age cohort being individuals aged 35-44 years (27.9%). A slight majority of respondents were married (55.0%) and reported having children (52.7%). In terms of educational attainment, most participants held a university degree (57.6%) or a postgraduate qualification (22.0%). White-collar employees constituted the largest occupational group (53.1%), and a substantial majority resided in urban areas (81.0%). It should be noted that, due to the non-probability nature of the sampling method, the findings are not generalizable to the broader population.

### Measures

Each scale included 5 items rated on a 5-point Likert scale (1 = strongly agree, 5 = strongly disagree), with lower scores indicating stronger agreement. Reverse coding was applied as needed to ensure internal consistency. A 2-week pilot study led to the removal of 2 items from the general vaccine attitude and trust scales. All measures were adapted from prior studies: general vaccine hesitancy,^31^ belief in conspiracy theories,^10^ trust in science and scientists,^19^ and coronavirus vaccine hesitancy.^32^ All scales exceed the recommended cutoff value of 0.70^33^ (see Table 1).

### Data Collection

Data were gathered through an online and paper-based survey using a non-probability snowball sampling method. A total of 469 adults participated.

### Ethics

Ethical approval for this study was obtained from the Ankara Hacı Bayram Veli University Ethics Committee (Date: February 01, 2024, Approval No: E-11054618-302.08.01-245007). Digital informed consent was gained from online participants, and written consent was secured from face-to-face participants.

### Statistical Analysis

Quantitative analyses were conducted using IBM SPSS (Statistical Package for the Social Sciences) Statistics version 26.0. In addition to descriptive statistics, mediation analyses were carried out using Hayes’ PROCESS macro (Model 4) to test IEs. Each model was run with 5000 bootstrap resamples to estimate 95% CIs. This approach allowed for the simultaneous testing of parallel mediation paths.

Table 2 shows Pearson correlations among key study variables. The average score for COVID-19 vaccine attitudes was 3.24 (SD = 1.05), and 3.20 (SD = 0.98) for general vaccine attitudes, both on a 5-point Likert scale (1 = strongly agree, 5 = strongly disagree), representing moderately favorable views. The Trust in Science scale had a mean of 2.48 (SD = 0.61), showing mostly high trust, as lower scores indicate stronger agreement. Belief in conspiracy theories averaged 2.99 (SD = 0.89), indicating neutral to slightly skeptical views. Overall, participants showed moderate support for vaccines and trust in science, with uncertainty toward conspiracies.

According to the Pearson Correlation analysis, general vaccine hesitancy was positively correlated with COVID-19 vaccine hesitancy (r = 0.567, *P* = .000), indicating consistent skepticism. Both were also positively correlated with Trust in Science scores (general: r = 0.237, COVID-19: r = 0.346; *P* = .000), indicating that greater hesitancy was related to lower trust, given that higher scores reflect less agreement. Belief in conspiracy theories was negatively correlated with trust (r = –0.166), general hesitancy (r = 0.578), and COVID-19 hesitancy (r = –0.495), showing that stronger conspiracy beliefs were associated with lower trust and higher hesitancy (see Table 2).

## Results

### Hypothesis Testing

To evaluate all hypotheses simultaneously, PROCESS Macro Model 4 was employed with bootstrap sampling and 95% CIs.^16^^,^^34^ Model 4 provides estimates of both direct and IEs. Since the model was tested holistically, direct and IEs were examined together.

As shown in Figure 1, trust in science and scientists has a significant positive direct effect on attitudes toward the COVID-19 vaccine (b = 0.37, se = 0.06, *P* = .000), supporting H1.

Additionally, Model 4 revealed that trust in science indirectly influences vaccine attitudes through 2 mediators: belief in conspiracy theories (IE = 0.0725; Bootstrap CI (BCI) = [0.0185, 0.1393]) and general vaccine attitudes (IE = 0.1540; BCI = [0.0757, 0.2510]). Because the BCIs for both IEs exclude zero, these variables are confirmed as simultaneous mediators. Therefore, H2 and H3 are supported.

To compare the 2 specific IEs, the “Contrast Indirect Effects” function of the PROCESS Macro was used. Since both IEs had the same sign (positive in this case), the default comparison method: the raw difference was used.^16^^,^^34^^,^^35^ The analysis showed that the difference between the 2 specific IEs was not statistically significant, as the bootstrap CI included zero (IE = −0.0815; BCI [−0.1814, 0.0006]). Although the effects differ numerically, this difference is not statistically meaningful. Therefore, H4 was not supported.

The results taken together back the suggested parallel mediation model: trust in science affects COVID-19 vaccine attitudes, employing both decreased belief in conspiracy theories and more favorable general vaccine attitudes. Although both IEs were significant, their difference was not. This indicates that both pathways substantially link scientific trust to vaccination acceptance; overall attitudes may hold greater significance.

## Discussion

Starting from correlation analyses, the results indicated mostly aligned with theoretical assumptions. First, general vaccine hesitancy and COVID-19 vaccine hesitancy was positively correlated with trust in science and scientists, while Belief in conspiracy theories was negatively correlated.^4^^,^^7^ The findings demonstrate that trust in science and scientists significantly influences vaccine hesitancy; however, the contradictory results reported by Salali and Uysal (2021) and Chayinska et al (2022) highlight the critical mediating role of general vaccine hesitancy and conspiracy beliefs in shaping public sentiment about the COVID-19 vaccine. In this context, it has been noted that even when individuals trust in science’s ability to uncover truth, a lack of analytical thinking skills combined with a tendency toward intuitive thinking^6^ may position conspiracy beliefs as a key mediating variable. Existing studies emphasize that a high level of analytical thinking ability reduces belief in conspiracy theories.^28^^,^^29^ Consequently, within this relational framework, the mediating effect of conspiracy theories and general vaccine attitudes becomes increasingly significant.

This study revealed that trust in science indirectly influences attitudes toward the COVID-19 vaccine through both beliefs in conspiracy theories and general vaccine hesitancy.^6^^,^^30^ Based on the results, the stronger beliefs in conspiracy theories were linked to reduced trust and increased vaccine hesitancy.^13^ The findings highlight the need for public health strategies that not only ensure access to accurate information but also actively combat misinformation and conspiracy narratives to increase trust in general vaccine acceptance.

Although the difference between the 2 mediators was not statistically significant,^9^ general vaccine hesitancy demonstrated a larger IE, suggesting a potentially stronger mediating role. This implies that broader attitudes toward vaccination, beyond those specific to COVID-19, may play a decisive role in shaping vaccine acceptance. Individuals’ past vaccination experiences and deeply held beliefs appear to influence their health-related decisions, even in times of crisis.^28^^,^^29^

In this context, health communication strategies should prioritize long-term approaches aimed at transforming general vaccine perceptions, rather than focusing solely on pandemic-specific messaging. Additionally, community-based programs that aim to strengthen public trust in scientists and promote transparent, evidence-based dialogue should be supported. In parallel with the study, Bozkurt (2023) and Freeman et al (2022) have concluded, based on scientific evidence, that the most effective antidote to vaccine hesitancy lies in fostering trust in scientists and doctors.

Finally, these findings hold value for evaluating generalizability across different sociocultural contexts and over time. Researchers should employ longitudinal designs to explore the stability and evolution of these mediating relationships in the future. Additionally, a limitation of this study is the omission of demographic variables (e.g., gender, age, income status, and education level). Therefore, it is recommended that future research investigate whether these variables contribute to differences in COVID-19 vaccine attitudes and trust in science and scientists. Additionally, focusing on areas that facilitate the spread of misinformation, such as social media usage practices (e.g., platform preferences, frequency of information sharing, and perceptions of credibility), would be beneficial for better understanding these dynamics and developing effective intervention strategies. However, the study’s use of non-probability sampling and cross-sectional data limits causal inference and generalizability to the broader population.

This study revealed that trust in science indirectly influences attitudes toward the COVID-19 vaccine through both beliefs in conspiracy theories and general vaccine hesitancy. The findings highlight the need for public health strategies that not only ensure access to accurate information but also actively combat misinformation and conspiracy narratives.

Although the difference between the 2 mediators was not statistically significant, general vaccine hesitancy demonstrated a larger IE, suggesting a potentially stronger mediating role. This implies that broader attitudes toward vaccination, beyond those specific to COVID-19, may play a decisive role in shaping vaccine acceptance. Individuals’ past vaccination experiences and deeply held beliefs appear to influence their health-related decisions, even in times of crisis.

In this context, health communication strategies should prioritize long-term approaches aimed at transforming general vaccine perceptions, rather than focusing solely on pandemic-specific messaging. Community-based programs that aim to strengthen public trust in scientists and promote transparent, evidence-based dialogue should be supported. Moreover, it is imperative that states reassess and adapt their communication strategies in accordance with the dynamics of the post-truth era. In an environment where information circulates rapidly and widely, the challenge is no longer limited to ensuring access to accurate data; rather, it also involves confronting the increasing ease with which information can be distorted, manipulated, and weaponized.

This study contributes to the literature by showing how trust in science influences vaccine acceptance through conspiracy beliefs and general vaccine attitudes.

Investigating these mediators simultaneously in Türkiye offers a nuanced view of how belief systems shape health behavior during global crises. However, the use of non-probability sampling limits causal inference and generalizability. Based on the findings of this study, future research should examine whether the IEs of general vaccine attitudes and belief in conspiracy theories differ by gender, being stronger or weaker among women compared to men, and by income level, particularly among those with lower versus higher income.

## Figures and Tables

**Table 1.** Measurement Properties

Constructs/Items	Alpha
“COVID-19 vaccine hesitancy scale (1 = strongly agree, 5 = strongly disagree)”	0.825
“COVID-19 vaccines are important for my health”	
“COVID-19 vaccines are effective”	
“Being vaccinated for COVID-19 is important for the health of others in my community”	
“The information I receive about COVID-19 vaccines from the government vaccine program is reliable and trustworthy”	
“I believe that vaccines should be compulsory during a pandemic”	
“I plan to do what my doctor or healthcare provider recommends about COVID-19 vaccines”	
“I am concerned about the serious adverse effects of COVID-19 vaccines”	
General vaccine attitudes (items 3 and 11 were reverse-coded as 5 = strongly agree, 1 = strongly disagree; all other items were coded as 1 = strongly agree, 5 = strongly disagree)	0.870
“If everyone gets vaccinated, diseases will decrease”	
“The strongest measure against pandemics is vaccination”	
“I am concerned about the side effects of vaccines	
“I am afraid that vaccines may cause autism or learning disabilities”	
“Vaccines can lead to many diseases”	
“Vaccines contain toxic substances”	
“I prefer gaining immunity by getting sick rather than being vaccinated”.	
“Vaccination should be voluntary, not mandatory”.	
“I avoid vaccines because I am afraid of needles”	
“Vaccines may cause permanent illnesses; therefore, I do not vaccinate my child”	
“Since pandemics are rarely seen, vaccination is unnecessary”	
Trust in science and scientists (Items 1 and 10 were reverse-coded as 5 = strongly agree, 1 = strongly disagree; all other items were coded as 1 = strongly agree, 5 = strongly disagree)	0.856
“When scientist change their mind about a scientific idea, it diminishes my trust in their work”	
“Scientists ignore evidence that contradicts their work”	
“Scientific theories are weak explanations”	
“I trust that the work of scientists to make life better for people”	
“We should trust in the work of scientists”	
“We should trust that scientist are being ethical in their work”	
“Scientific theories are trustworthy”	
“Science provides explanatory answers about our world”	
“I believe scientists can find solutions to our major health problems”	
“Scientists do not value the ideas of others. Today’s scientists will sacrifice the well-being of others to advance their research”	
Belief in conspiracy theories (1 = strongly agree, 5 = strongly disagree)	0.908
“Coronavirus is a fraud”	
“The coronavirus was created in a laboratory”	
“Coronavirus was developed so that governments can seize political control”	
“Coronavirus is being spread via 5G technology”	
“Bill Gates and his foundation are behind the coronavirus”	
“The antibody test is a conspiracy to collect our DNA”	

**Table 2.** Pearson Correlations Among Key Study Variables

Total Sample	Mean	Standard Deviation	1	2	3	4
1. General vaccine hesitancy	3.24	1.049	1			
2. Covid-19 Vaccine hesitancy	3.20	0.981	0.567^**^	1		
3. Trust in science	2.48	0.605	0.237^**^	0.346^**^	1	
4. Conspiracy theory (belief)	2.99	890	−0.578^**^	−0.495^**^	−0.166^**^	1

*P < .05.

**P < .01.

***P < .001.

## Data Availability

The data that support the findings of this study are available on request from the corresponding author.
